# Dynamin-Related Protein 1 at the Crossroads of Cancer

**DOI:** 10.3390/genes9020115

**Published:** 2018-02-21

**Authors:** Ana Rita Lima, Liliana Santos, Marcelo Correia, Paula Soares, Manuel Sobrinho-Simões, Miguel Melo, Valdemar Máximo

**Affiliations:** 1 Cancer Signaling & Metabolism Group, Instituto de Investigação e Inovação em Saúde (Institute for Research and Innovation in Health Sciences) (I3S), University of Porto, 4200-135 Porto, Portugal; arlima@ipatimup.pt (A.R.L.); lsantos@ipatimup.pt (L.S.); mcorreia@ipatimup.pt (M.C.); psoares@ipatimup.pt (P.S.); ssimoes@ipatimup.pt (M.S.-S.); jmiguelmelo@live.com.pt (M.M.); 2 Cancer Signaling & Metabolism Group, Institute of Molecular Pathology and Immunology of the University of Porto (IPATIMUP), 4200-135 Porto, Portugal; 3 Medical Faculty of University of Porto—FMUP, 4200-135 Porto, Portugal; 4 Abel Salazar Biomedical Sciences Institute (ICBAS), University of Porto, 4200-135 Porto, Portugal; 5 Department of Pathology, Medical Faculty of University of Porto (FMUP), 4200-135 Porto, Portugal; 6 Department of Pathology and Oncology, Centro Hospitalar São João, 4200-135 Porto, Portugal; 7 Department of Endocrinology, Diabetes and Metabolism, Centro Hospitalar e Universitário de Coimbra (Coimbra University Hospital Centre), 3000-075 Coimbra, Portugal; 8 Faculty of Medicine, University of Coimbra, 3000-548 Coimbra, Portugal

**Keywords:** dynamin-related protein 1, mitochondrial biogenesis, tumorigenesis, cancer, metabolism

## Abstract

Mitochondrial dynamics are known to have an important role in so-called age-related diseases, including cancer. Mitochondria is an organelle involved in many key cellular functions and responds to physiologic or stress stimuli by adapting its structure and function. Perhaps the most important structural changes involve mitochondrial dynamics (fission and fusion), which occur in normal cells as well as in cells under dysregulation, such as cancer cells. Dynamin-related protein 1 (DRP1), a member of the dynamin family of guanosine triphosphatases (GTPases), is the key component of mitochondrial fission machinery. Dynamin-related protein 1 is associated with different cell processes such as apoptosis, mitochondrial biogenesis, mitophagy, metabolism, and cell proliferation, differentiation, and transformation. The role of DRP1 in tumorigenesis may seem to be paradoxical, since mitochondrial fission is a key mediator of two very different processes, cellular apoptosis and cell mitosis. Dynamin-related protein 1 has been associated with the development of distinct human cancers, including changes in mitochondrial energetics and cellular metabolism, cell proliferation, and stem cell maintenance, invasion, and promotion of metastases. However, the underlying mechanism for this association is still being explored. Herein, we review the published knowledge on the role of DRP1 in cancer, exploring its interaction with different biological processes in the tumorigenesis context.

## 1. Introduction

Mitochondrial dynamics is known to have an important role in the so-called age-related diseases, including obesity and type 2 diabetes, Parkinson’s disease, Alzheimer’s disease (AD), and cancer. Despite this, research on cancer and mitochondrial dynamics has only recently started to be unveiled [1,2,3,4].

Mitochondria are organelles involved in many key cellular functions, such as adenosine triphosphate (ATP) production, cell anabolic and catabolic functions, calcium signaling, cell division and differentiation, and cell death [5,6,7]. Mitochondria respond to physiologic or stress stimuli by adapting their structure and function, which are intimately connected [8]. In recent years, much has been explored on the key molecules and processes that intervene on, or drive, some of these structural and functional changes. Perhaps the most important of such structural changes is the phenomena of mitochondrial fission and fusion, which occur in normal cells, as well as in cells under dysregulation, such as cancer cells, as reviewed by Chen and Chan, and Westermann [9,10]. Mitochondrial fission secures an adequate number of mitochondria to support growing and dividing cells [8,9]. Mitochondrial fission also generates new organelles and represents a quality control mechanism by eliminating damaged mitochondria through selective autophagy, also called mitophagy [9,11]. Mitochondria fusion, on the other hand, is required for maximal ATP production when mitochondria need to rely on oxidative phosphorylation, or when they have to react to stress stimuli, in which case they appear as elongated *healthy* organelles that complement the dysfunctional mitochondria [12,13,14]. Fusion also allows the exchange of proteins, metabolites and mitochondrial DNA (mtDNA) within the mitochondrial network, avoiding the accumulation of damaged contents in mitochondria [12,15]. Interestingly, Kowald and Kirkwood have proposed mitochondrial fusion as being a permissive mechanism to clonal expansion of mitochondrial deletion mutants, rather than a rescue mechanism for damaged mitochondria [16,17].

Dynamin-related protein 1 (DRP1), a member of the dynamin family of guanosine triphosphatases (GTPases), is the key component of the mitochondrial fission machinery [18]. Dynamin-related protein 1 has been linked to the development of different malignant tumors, including skin, brain, breast, lung, thyroid and endometrial cancer. However, the underlying mechanism(s) for this association is still being explored [19,20,21,22,23,24]. Dynamin-related protein 1 had roles in changing cellular metabolism in melanoma, contributing to stemness in glioblastoma, involvement with lymph node metastases in breast cancer, sustaining cell cycle and proliferation in lung cancer, and associations with the oncocytic phenotype in thyroid cancer [19,20,21,22,23]. Besides its impact on metabolic regulation, DRP1 has also been associated with a broad range of cell processes: apoptosis, mitochondrial biogenesis and mitophagy, cell proliferation, and differentiation and transformation [19,25,26,27,28,29].

Herein, we review the published knowledge on the role of DRP1 in cancer, exploring its interactions with different biological processes, particularly in the tumorigenesis context. Given the broad range of cellular processes where DRP1 is involved, and its interactions with key known hallmarks of cancer, we will start by reviewing DRP1 role in mitochondria fission and its regulation. Following this, we will provide an overview of DRP1 interplay with biological processes known to be altered in cancer which are important for tumor progression, such as cell death, metabolic programming, and the cell cycle (Table 1). We will then discuss dysregulation of these processes in different tumor models centered on DRP1 alterations, particularly the role of this protein in the invasion and metastization processes, relevant for the generalization stages of tumorigenesis. We will finish with a summary of future perspectives and potential clinical implications of targeting DRP1.

## 2. Regulation of Dynamin-Related Protein 1 and Its Central Role in Mitochondrial Fission

Mitochondrial fusion and fission proteins, first identified in flies and yeast, are key players in mitochondrial biogenesis [30]. There are three highly conserved dynamin-related GTPases (DRPs) regulating membrane dynamics in various cellular processes. These large proteins contain a canonical GTPase domain and various regions that enhance self-assembly via both intra- and inter-molecular interactions [31]. The mitochondrial fission components were first described in yeast genetic screening studies [32]. Dynamin 1 protein (Dnm1) is structurally related to the large dynamin family and was the first protein to have shown a clear role in controlling mitochondrial fission and morphology in *Saccharomyces cerevisiae* [33,34]. In 1998, Otsuga et al. have shown, in yeast, that dynamin-1-like gene (*DNML1*) mutants, with defects in the predicted GTP-binding domain, had a markedly distorted mitochondrial morphology and an altered network distribution, associated with the impairment of mitochondrial fission [33,34]. Around the same time, the human ortholog of dynamin-1-like protein (DNML1)- DRP1 - was described and was shown to be essential, and the main driver for mitochondrial division in mammalian cells [35,36].

Although DRP1 is described as being mostly a cytoplasmic protein, it has been detected both in cytosol and mitochondria in baseline conditions [19,20,21,22,23]. Indeed, DRP1 translocates to mitochondria upon activation of a stimulus, such as mitochondrial membrane uncoupling, where it links to receptors such as mitochondrial fission factor (MFF) and fission 1 protein (FIS1), constricting the outer mitochondrial membrane in a process dependent on GTPase activity [17]. While MFF is required for DRP1 recruitment, it should be noted that different studies have questioned the role of FIS1 in inducing mitochondrial fission [35,36,37,38,39]. Depending on the cell types and conditions other proteins, such as mitochondrial protein of 18 kDa (MTP18), ganglioside-induced differentiation-associated protein 1 (GDAP1), mitochondrial dynamics protein of 49 kDa and 51 kDa (MiD49 and MiD51), or mitochondrial elongation factor 1 (MIEF1) have a role in cytoplasmically-localized DRP1 activation needed for its recruitment to mitochondria fission sites [38,40,41]. Ganglioside-induced differentiation-associated protein 1 is mainly expressed in neurons and Schwann cells [42]. Additionally, endophilin was reported to act downstream of DRP1 and to be important in the maintenance of mitochondrial morphology [43].

**Figure 1:**
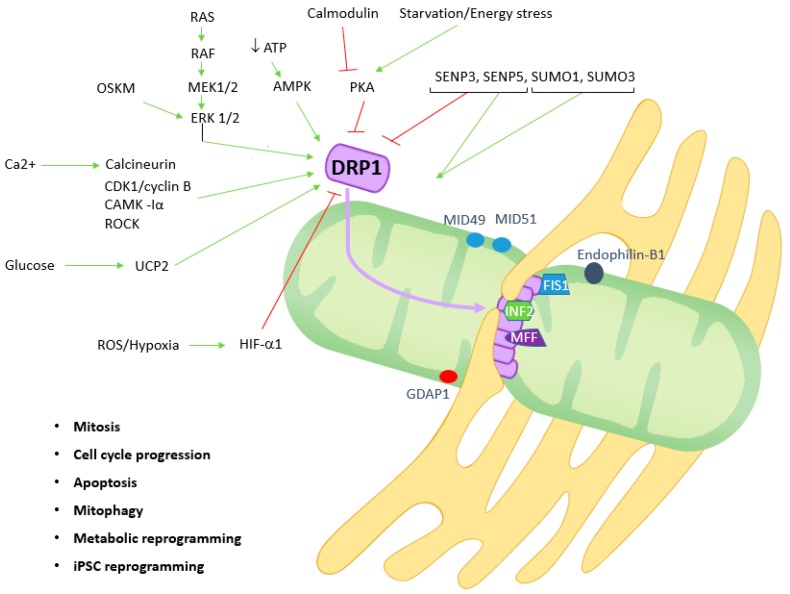
Key players and stimuli in DRP1-mediated mitochondrial fission, both in physiologic and tumor conditions. Green arrows represent stimulation or activation of pathway; red arrows represent repression or inactivation of pathway. SUMO1/Sentrin/SMT3 specific peptidase 3 and 5 (SENP3 and SENP5) and small ubiquitin-like modifier and small ubiquitin-like modifier 1 (SUMO and SUMO1). SENP are deSUMOylating enzymes. For a more in-depth review of the fission and fusion machinery please refer to Silva et al. [17].

Dynamin-related protein 1 assembles in spirals at sites where endoplasmic reticulum tubules cross over mitochondria and subsequent actin polymerization by inverted formin-2 (INF2) occurs, ultimately leading to mitochondrial fission, as depicted in Figure 1 [44]. Since localization of DRP1 and MFF is dependent on nucleoids, known to be structures composed of both mtDNA and proteins putatively involved in the replication of mtDNA, mitochondrial fission often occurs adjacent to nucleoids [45].

Of note, DRP1 overexpression does not lead to mitochondrial fission *per se*, since DRP1 activity is dependent on its activation by different post-translational modifications, and on the translocation from cytosol to mitochondria. These modifications may include phosphorylation, SUMOylation, ubiquitination, *S*-Nitrosylation and *O*-GlucNAcylation [46,47,48,49]. This fact should be kept in mind when interpreting the data described in the literature. The translocation of DRP1 from cytosol to mitochondria may also be impaired by GTPase domain mutations leading to defects in higher-ordered assembly [50]. Several kinases control DRP1 activity by phosphorylation at 3 main sites—Ser616, Ser637 and Ser693 [49,51,52,53,54,55,56]. The phosphorylation of DRP1^S616^ can be made by different protein kinases involved in signaling pathways, cell cycle, cell cytoskeleton, or Ca^2+^ signaling. These include protein kinase C (PKC), CDK1/Cyclin B in the context of mitosis, rho-associated coiled-coil kinase (ROCK) or Ca^2+^/calmodulin-dependent protein kinase (CAMK -Iα), to promote fission [51,54,57]. On the other hand, phosphorylation of DRP1^S637^, namely by protein kinase A (PKA), inhibits fission [51,52,53]. Opposite to this, dephosphorylation of DRP1^S637^ by calcineurin, which is activated by mitochondrial depolarization and by sustained cytosolic calcium increase, including in situations of starvation and apoptosis stimuli, promotes mitochondrial fission [57]. Finally, phosphorylation of DRP1^S693^ by glycogen synthase kinase 3β (GSK3β), a negative regulator of glycogenesis and a known regulator of various signaling pathways and cellular functions, has been demonstrated to prevent fission during apoptosis [49]. Several cancer signaling pathways involving PKA, AMP-activated protein kinase (AMPK) and epidermal growth factor receptor-retrovirus associated sequence oncogene signaling pathway (EGFR-RAS) activate DRP1 driven mitochondrial fission, as will be discussed later [19,28,29,58,59,60,61]. On the other hand, after induction of macroautophagy by starvation, mitochondria elongate both in vitro and in vivo [62]. Starvation induces an increase in cyclic adenosine monophosphate (cAMP) levels and leads to PKA activation which contributes to a more effective ATP production through mitochondria elongation [63]. For a more in-depth review of the fission and fusion machinery please refer to Silva et al. [17].

## 3. Dynamin-Related Protein 1 and Cell Death

Mitochondrial division and fusion regulate mitochondrial-dependent intrinsic apoptosis, which relies on the mitochondrial outer membrane permeabilization (MOMP) and in mediators of cell death, such as cytochrome *c*, to be released from the mitochondria [66,75,76,77,78]. Mitochondrial fusion protects cells from apoptosis driven by the role of optic atrophy 1 protein (OPA1) in cristae maintenance, which attenuates the MOMP-induced release of cytochrome *c* [79,80,81,82,83]. Mitochondrial fragmentation is known to be involved in several apoptotic models [65]. The role of DRP1 has been detected in complexes with bcl-2-associated X protein (BAX) at mitochondrial fission sites, contributing for the permeabilization of the outer mitochondrial membrane (OMM) and cytochrome *c* release [64]. The role of DRP1 in apoptosis and cell death, as in many other cell biological functions, may seem counterintuitive. Szabadkai et al. have used HeLa cells to overexpress DRP1 and thereby assess the role of mitochondrial division in apoptotic signaling and sub-cellular Ca^2+^ homeostasis [65]. The authors have observed a fragmentation of the mitochondrial network, and a blockage of the intramitochondrial Ca^2+^-propagating waves [65]. However, the apoptotic effect of ceramide on DRP1 expressing cells was significantly reduced, while sensitivity to staurosporine-induced apoptosis was enhanced, raising the hypothesis that a balance between fusion and fission processes may impact on mitochondrial Ca^2+^ responses [65]. In fact, ceramide acts by inducing Ca^2+^ release from the endoplasmic reticulum (ER) and also to sensitize mitochondria to Ca^2+^ impulse, while staurosporine has a direct effect on the OMM permeabilization [65]. Based on these findings, Szabadkai et al. proposed a model in which DRP1-mediated mitochondrial fission leads to mitochondria positioning far from the ER, thereby reducing the efficiency of Ca^2+^ uptake, which may still be sufficient for normal mitochondrial function, but may serve as a protective mechanism in responses to stress, preventing apoptosis [64]. Other studies have shown that the downregulation or knock-down of DRP1, or the use of mitochondrial division 1 inhibitor (Mdivi-1), widely used as putative specific DRP1 inhibitor, can prevent cell death and/or promote cell proliferation [22,66,67]. The interpretation of the data published using this compound should take into consideration the caveat of Mdivi-1 not being currently considered as a specific DRP1 inhibitor, but rather as a weak and reversible complex I inhibitor [84]. In particular, Cassidy et al. found that Mdivi-1, retards apoptosis by inhibiting mitochondrial OMM permeabilization and consequently cytochrome *c* release [66]. Rehman et al. have showed that the genetic inhibition, and the use of Mdivi-1, in human lung cancer cell lines led to a decrease in mitochondria fragmentation and a three- to four-fold increase in apoptosis [22]. Finally, Yamauchi-Inoue and Oda have demonstrated that DRP1 knockdown in human colon cancer cells resulted in significantly reduced proliferation, increased percentage of cells in sub-G_0_/G_1_ cell cycle phase, caspase-3 activation and apoptosis [67]. Interestingly, a reduction in mitochondrial membrane potential was also observed, which may explain the release of cytochrome *c* seen in apoptosis following caspase activation [67].

All this evidence highlights the potential dual role of DRP1 on cell death and cell proliferation. On one hand, DRP1 may act as a gatekeeper, preventing apoptosis under sub-maximum stress conditions; on the other DRP1-driven mitochondrial fission is needed for cell death and cell proliferation to occur, as explained before. These opposing effects will also become obvious in the tumorigenesis section below, where DRP1 expression or activity may reflect pro-apoptotic or pro-proliferative traits, the former being potentially advantageous for therapeutic purposes.

## 4. Dynamin-Related Protein 1 and Metabolic Reprogramming

The relationship between mitochondrial morphology and cell energetics and survival has already been documented. Mitochondrial elongation increases mitochondrial function and protects cells from apoptosis [62,85]. Cells tend to present mitochondria in an elongated form under starvation conditions, and in a fragmented state under a nutrient-rich environment [62,85]. Mitochondrial elongation contributes to mitochondrial function and protects cells from apoptosis under conditions of starvation in mouse embryonic fibroblasts (MEF) cells [62,86]. Mitochondrial metabolic reprogramming is a hallmark of tumorigenesis, and it has been well described that in most of the tumor cell types, an increase in aerobic glycolysis takes place, a phenomenon known as the Warburg effect [87]. However, it is also recognized that cancer cells can adapt their metabolic profile to their needs. A study that shed light on how mitochondrial morphology links with metabolism plasticity in cancer cells was published by Li et al., who have investigated the changes in mitochondrial morphology induced by nutrition deprivation in tumor cells, using different tumor type cell lines [70]. A dramatic mitochondrial elongation was induced by starvation. This finding was concomitantly associated with a significant decrease in the DRP1 mitochondrial fraction and a dramatic increase in the phosphorylated form DRP1^S637^ driven by PKA activation, proven to be required for the energy stress-induced mitochondrial elongation in hepatocellular cell carcinoma (HCC) cell lines [70]. More importantly, mitochondrial elongation was found to induce a metabolic shift from glycolysis to oxidative phosphorylation during energy stress [70]. Mitochondrial elongation induced by energy stress facilitated cristae formation and the assembly of respiratory chain complexes I–IV to promote oxidative phosphorylation [70]. This, in its turn, led to a negative feedback effect on glycolysis through nicotinamide adenine dinucleotide (NAD^+^)-dependent sirtuin 1 (SIRT1) activation, a nutrient-sensing deacetylase [70]. Starvation treatment inhibited the acetylation of hypoxia-inducible factor 1α (HIF-1α) and the expression of pyruvate dehydrogenase kinase 1 (PDK1) and lactate dehydrogenase A (LDH-A), which are known to be HIF-1α target genes. This was reversed by the expression of the mutant DRP1^S637A^, which was associated with mitochondrial fission [70]. This study also indicated that DRP1^S637^-mediated mitochondrial elongation also predicted a poor prognosis in hepatocellular carcinoma patients [70]. Expression of phosphorylated DRP1^S637^ was found to be significantly correlated with larger tumor size, high tumor-node metastasis stage, and a significantly reduced overall survival and recurrence free survival [70]. Consistent with these results, nutrient deprivation was associated with OXPHOS/glycolysis interchange in a human glioma cell line, via HIF-1α/cellular myelocytomatosis oncogene protein (c-MYC) pathway, although a correlation with potential changes in mitochondrial shape has not been assessed in this study [71]. Interestingly, metabolic reprogramming is also a finding that seems to be associated with precancerous lesions of the colon, where a significant increase in gene expression of *DNML1* was shown, which was accompanied by indirect markers of the Warburg effect in human samples, as reported by Cruz MD et al. [88]. Zou et al. have elucidated how DRP1 dysregulation may interact with mitochondrial biogenesis and mitochondrial autophagy (mitophagy), and thereby with metabolic reprogramming. The authors have assessed the autophagic flux by evaluating the impact of autolysosome inhibitors on the microtubule-associated protein-1 light chain 3α phosphatidylethanolamine conjugate (LC3-II) levels, a protein known to be important for autophagosome formation [68]. They have shown a pattern of DRP1 upregulation, which was associated with metabolically less active mitochondria in a breast cancer cell line. This was accompanied by a reduction in the number of mitochondria, an increase of mitochondrial biogenesis markers such as peroxisome proliferator-activated receptor γ coactivator 1-α (PGC1α), nuclear respiratory factor 1 (NRF1), and mammalian mitochondrial transcription factor (TFAM), and a significant upregulation of B-cell lymphoma 2 protein (BCL-2) nineteen-kilodalton interacting protein 3 (BNIP3), a mitophagy marker, and of the autophagic flux, suggesting an increased mitophagy that explained the reduced number of mitochondria [68]. This pattern was also confirmed in vivo in human breast carcinoma tissue, based on the analyses of a series of human breast cancer from The Cancer Genome Atlas (TCGA database) [68]. Breast cancer cell lines exposed to Mdivi-1 exhibited a reduced autophagic flux and a shift from a glycolytic to an oxidative phenotype, suggesting a reversal of the Warburg effect [68]. The authors suggested a role of DRP1 in the coordinated increase of mitochondrial biogenesis and mitophagy, and in the regulation of breast cancer cell metabolism and survival since a significant decrease of cancer cell viability was also shown. It would be interesting to assess whether these Mdivi-1-induced metabolic effects can be explained by DRP1 inhibition, or through its currently proposed mechanism of action as a reversible Complex I inhibitor [84].

Beyond the effects of starvation in the metabolism of cancer cells, it is also of the utmost relevance to explore the role of hypoxia on metabolic tumor cell adaptation. Using mtDNA-enriched (SK-N-AS) and depleted (ρ0) cells of neuroblastoma cultured in hypoxic conditions, Kuo et al. have shown that hypoxia-stimulated HIF-1α expression, which was also influenced by the level of reactive oxygen species (ROS), was accompanied by increases of LDH-A and PDK1 as well as an increased expression of DRP1 [69]. Additionally, in mtDNA-enriched cells, a higher expression of DRP1 during hypoxia was observed, which was reverted after genetic suppression of HIF-1α [69]. Indeed, mtDNA seemed to be a mediator of HIF-1α, linking metabolic reprogramming to mitochondrial biogenesis [69].

All these data underscore the role of DRP1 as an indirect mediator of a metabolic shift under starvation conditions, when cancer cells need to rely on a more efficient energy production process (OXPHOS) as opposed to the classic glycolytic phenotype. On the other hand, DRP1 should also be seen as a key linking piece that connects different features of the same process (metabolic reprogramming, to meet cell energy needs, mitochondrial biogenesis, building the cell powerhouse that delivers that energy, and mitophagy, a system that promotes the quality control of mitochondria, as will be seen later). Therefore, depending on the different stimuli and needs, and even depending on specific driver oncogenes, the role of DRP1 is possibly two-pronged: being permissive to OXPHOS or promoting glycolysis.

## 5. Dynamin-Related Protein 1 and the Cell Cycle

Mitochondrial fission occurs during cellular division, thus securing a proper mitochondrial number in daughter cells. Dynamin-related protein 1 has been described to be functionally or molecularly linked to Cyclin B, E and D [19,29,54,55,72,73]. As previously mentioned, mitochondrial fission during mitosis depends on translocation of DRP1 to mitochondria and phosphorylation of DRP1^S616^ by Cyclin B-CDK1 [89]. On the other hand, mitochondrial shape was found to regulate the cell cycle, as demonstrated by the relationship between the mitochondrial hyperfusion at G_1_-S and the Cyclin E buildup needed to entry into S phase [89]. Additionally, DRP1 has been identified as one of the Cyclin D1-interacting proteins in human tumors, including breast and colorectal cancer [89]. Previous studies have demonstrated that DRP1-driven mitochondrial fission is critical for regulation of cell proliferation in a *Drosophila* model system, as well as in mammalian cells [89]. Mitochondrial function can impact cell cycle regulation; however, this has been an underexplored area in cancer research.

Taguchi et al. have studied mitochondrial dynamics and inheritance in mammalian cells undergoing mitosis in vitro and they showed that mitochondria have a tubular network appearance in interphase cells, moving into fragmented status in early mitotic stage, and going back to filamentous structures in the late phase of mitosis, the mitochondrial fission being a result of DRP1^S585^ phosphorylation by CDK1/Cyclin B [54]. Although the exact mechanism by which fission occurs is not yet totally known, endophilin and probably other downstream factors may play a role [90]. The elongated shape of mitochondria in G_1_/S interface is linked to the cellular requirement of ATP and high Cyclin E levels [29,72]. It is therefore thought that throughout the cell cycle, mitochondrial dynamics provides the energy requirements that are needed.

Parone et al. showed that downregulation of DRP1 in HeLa cell lines causes mitochondrial dysfunction, with an increase in ROS levels, a loss of mtDNA, a reduction in cellular ATP, proliferation arrest, and autophagy [91]. It seems therefore that cellular homeostasis is dependent on DRP1-dependent mitochondrial fission. On the other hand, mitochondrial hyperfusion induced by DRP1 deficiency was found to trigger a signaling of replicative stress by which ataxia-telangiectasia mutated/checkpoint kinases 2 and 1 (ATM/Chk2 and ATR/Chk1) DNA damage signaling pathways, as well as the ATM kinase-dependent G_2_/M cell cycle checkpoint, are activated [72]. A pattern of genomic instability and aneuploidy in p53 wild type and mutated cells, independent of ATP production defects or ROS production, was also found, suggesting that DRP1 may be implicated in mitochondria-nucleus retrograde signaling and raising the hypothesis that mitochondria play a role in tumorigenesis [72].

Rehman et al. have compared the level of mitochondria fragmentation in several human lung cancer cell lines and normal human cell lines. They observed that all malignant cells presented a markedly higher level of mitochondria fragmentation, which was linked to higher DRP1 and lower mitofusin-2 (MFN2) expression levels, the latter being a protein involved in mitochondrial fusion [22]. The same was observed in lung adenocarcinoma samples, when compared to adjacent normal lung tissue. Additionally, the levels of phosphorylated DRP1^S616^ were also significantly higher, as opposed to phosphorylated DRP1^S637^ which was lower in both lines. The genetic inhibition of DRP1, and the use of Mdivi-1, has led to a decrease in mitochondrial membrane potential, and a decrease in the number of cells in S phase and an increase in number of cells in G_2_ phase, again indicating an inhibition of the mitotic program [22]. Both these interventions were also tested in a lung adenocarcinoma xenograft model, leading to a significant decrease in tumor size [22].

Mitra et al. have reported a relationship between mitochondrial morphology and cell cycle control at the G_1_–S boundary [29]. Mitochondria change from fragmented structures into a hyperfused state at G_1_–S transition. In this stage of the cell cycle, the mitochondrial network presents a greater ATP output than isolated mitochondria at any other cell cycle stage. Hyperfused mitochondria might also play a role in tumorigenesis, since it is known that many cancer cells present dysregulated Cyclin E levels, the cyclin responsible for G_1_-to-S phase progression and lose control of G_1_–S transition [92,93].

Zhan et al. have shown that the expression of DRP1 increased mitochondrial fission and promoted the proliferation of HCC cells both in vitro and in vivo, by enhancing the G_1_/S phase transition [94]. Additionally, the authors have verified that DRP1 knockdown induced a significant G_1_ phase arrest in vitro, and reduced tumor growth in vivo [94]. More importantly, they have demonstrated that the promotion of proliferation by DRP1-mediated mitochondrial fission was mediated through p53/p21 and nuclear factor kappa B (NF-κB) /cyclins pathways [94].

Finally, Tanwar et al. have recently published an exploratory analysis of gene expression data from the 31 cancer types in TCGA, showing that DRP1 is predominantly co-expressed with genes involved in the cell cycle, and in gene expression and metabolism, across the majority of the cancer types [74]. In particular, their investigation on epithelial ovarian cancer (EOC) revealed that DRP1 co-expresses with the cell-cycle module responsible for mitotic transition, which included over 70 genes involved in various phases of cell cycle (G_1_ phase, G_1_/S transition, S phase, G_2_/M transition and M), such as the mitotic transcription factor forkhead box M1 (FoxM1) and its key downstream targets regulating mitotic transition. Inactivation of DRP1 through DRP1 knock-down in EOC cells led to attenuation in mitotic transition [74]. Interestingly, DRP1-cell-cycle co-expression module was detected in epithelial ovarian tumors which responded to chemotherapy, suggesting that DRP1 driven mitosis may contribute to chemo-sensitivity of the primary tumors.

In summary, the pattern of higher DRP1 expression observed in different malignant tumors, as we will later see, seem to indicate a higher proliferative profile of those cells. Complementary to this, DRP1 could also represent a caretaker mechanism, in the sense that its downregulation can trigger the activation of DNA damage signaling pathways, and in an extreme context, ultimately lead to tumorigenesis. The fact that DRP1 is directly involved in cell cycle progression makes it an attractive target for directing therapy agents that interfere with cell proliferation.

## 6. Dynamin-Related Protein 1 Expression and its Role in Tumorigenesis

DRP1 expression patterns and its role in cancer have been documented in several tumor models and are summarized in Table 2. Wieder et al. described an expression of phosphorylated DRP1^S616^ in nearly half of the cases of a melanoma series, 95.6% of which were *BRAF^V600E^* tumors [19]. Interestingly, the same relationship with B-Raf proto-oncogene (*BRAF*) status was observed in dysplastic nevi, with 92% of *BRAF^V600E^* samples being positive for phosphorylated DRP1^S616^ [95]. Genetic inhibition of DRP1 in *BRAF^V600E^* melanoma cell line led to a loss of expression of DRP1 that was correlated with decreased cell proliferation. On the other hand, the use of Mdivi-1 led to a decrease in DRP1-dependent mitochondrial fission and dose-dependent apoptosis, which was not seen in the wild type (WT) *BRAF^WT^* melanoma cell line, suggesting that the induction of phosphorylated DRP1^S616^ in dysplastic nevi and in primary melanoma may be a contributing factor to *BRAF^V600E^* disease, raising the question of its potential role as a prognosis biomarker in this context [95]. These results should take into consideration the caveat of Mdivi-1 not being currently considered a specific DRP1 inhibitor [84,95].

Rehman et al. documented an increase in DRP1 expression in tissue samples from patients with lung adenocarcinoma [22]. An identical pattern was observed in cultured lung cancer cell lines, with increased levels of phosphorylated DRP1^S616^ and decreased levels of phosphorylated DRP1^S637^ [22]. Interestingly, Mdivi-1 was tested in a lung adenocarcinoma xenograft model and proved to significantly reduce tumor size, with an increase in the uptake of 18F-fluorodeoxyglucose (^18^FDG) in the residual tumor, suggesting an effect on tumor metabolism [22]. Considering the currently proposed mechanism of action of Mdivi-1, as an inhibitor of complex-I and ROS production, it would be interesting to assess if the described reduction of tumor size may be related with potential changes in mitochondrial metabolism.

Ferreira-da-Silva et al. studied benign and malign thyroid tumors, including oncocytomas, which are characterized by a large accumulation of mitochondria in the cytoplasm of their cells [23,99,100]. Interestingly, they found a statistically significant overexpression of DRP1 protein in the oncocytic versus the non-oncocytic thyroid tumors. This pattern was also found when they compared oncocytic carcinomas with oncocytic adenomas [23]. However, the same trend was not observed when comparing benign and malignant tumors overall, nor within the non-oncocytic group of adenomas versus carcinomas. Following these same findings, Ferreira-da-Silva et al. have documented a statistically significant higher expression of DRP1 in an oncocytic thyroid carcinoma cell line compared with a non-oncocytic cell line, an observation that was not explained by differences in mRNA expression [23]. The higher expression of DRP1 was also associated with a more fragmented mitochondrial network [23]. The genetic inhibition of DRP1 reduced cell motility in the oncocytic cell line by close to 50%, a pattern that was also seen with the use of Mdivi-1 [23]. The higher DRP1 expression and fission profile may explain the oncocytic pattern of this particular subset of thyroid tumors, given the known role of DRP1 in mitochondrial biogenesis [23,101]. The association between DRP1 and the potential for higher migration and invasion capacities of the malignant oncocytic tumors is a trait that may also be explained by DRP1 overexpression, and one that has been shown in other tumor models, as later described [23].

Serasinghe et al. have shown that E1A plus *RAS^G12V^*-infected MEFs induce DRP1 mRNA expression, DRP1 expression, its activation through phosphorylation of serine 952 residue (murine equivalent of DRP1^S616^ phosphorylation), and a glycolytic phenotype [19]. Through *DRP1* genetic inhibition, and also after the use of Mdivi-1, DRP1 expression and function were found to be required for MAPK/ERK kinase (MEK) triggered transformation when *RAS^G12V^* signaling is induced [19]. When they tested two small MEK inhibitors in those transformed cells this led to increased mitochondrial fusion, which was shown to be directly related to the phosphorylation of DRP1^S592^ [19]. Similar results were observed in a human *BRAF^V600E^* melanoma cell line, where different upstream and downstream mitogen-activated protein kinase (MAPK) inhibitors have led to mitochondrial fusion, which seemed to be dependent on direct effects in the MAPK pathway, since drug-resistant cell lines were not sensitive to this effect [19]. This result seemed to be independent of mitochondrial biogenesis and was reversible [19]. Similarly, MAPK inhibitors inhibited DRP1 mRNA, protein, and DRP1^S616^ phosphorylation, and led to reprogramming of mitochondrial metabolic function, shifting it to an OXPHOS patterned metabolism [19]. These authors also documented a significantly higher phosphorylated DRP1^S616^ expression rate in *BRAF^V600E^* melanoma patient samples when compared with *BRAF^WT^* samples [19]. According to Serasinghe et al. experiments, DRP1 seems to regulate mitochondrial function before an oncogenic signaling is initiated, during carcinogenesis and after oncogenic MAPK signaling inhibition [19].

Lennon et al. have specifically explored mitochondrial morphology through fractal dimension and lacunarity measurements in mesothelioma cell lines, as a prediction of responses to treatments that interfere with mitochondrial metabolism [102]. Fractal dimension and lacunarity are quantitative measurements which allow the description of complex structures, such as mitochondria. The former relies on a mathematical principle of self-similarity between different biological structures, while the latter is based on the texture of a shape. An altered ratio of DRP1-MFN2 in both total cell lysates and mitochondrial fraction was detected, suggesting a higher relative rate of fission as compared to fusion [102]. Interestingly, mitochondrial morphology showed a better correlation with mitochondrial inhibitors sensitivity than did metabolic function [102]. As pointed out by the authors, increased fission seemed to be associated with decreased mitochondrial activity and mitochondrial membrane potential, which could explain an increase in cell death with mitochondrial inhibitors [102].

Hagenbuchner et al. have studied the mitochondrial effects of Survivin, a known anti-apoptotic protein that is overexpressed in neuroblastoma with gain of chromosome 17q, typically associated with high stage cancer, poor prognosis, and chemotherapy resistance [97]. In Survivin-expressing cells, mitochondria presented as punctuated, perinuclear structures, due to an increase in the expression of DRP1, which was accompanied by a reduction in the expression of BCL-2-like protein 11 (BIM) [97]. In these cells, DRP1 localized in mitochondria, but no cytochrome *c* release was observed due to the absence of BIM [97]. These effects were affected through genetic inhibition of DRP1, and also after the used of Mdivi-1 [97]. Curiously, an effect of Survivin on oxidative phosphorylation, through an impact on complex I and IV, was also shown to result from DRP1-induced mitochondrial fission, with no changes in ATP levels, raising the hypothesis that ATP in these Survivin expressing cells may be produced as a result of glycolysis, which was supported by the increase in glucose consumption and lactate production, and by the effect that glycolysis inhibitors had on cell viability reduction and sensitivity to chemotherapy agents [97].

Recently, Guerra et al. have documented an increase in the expression of DRP1 and BNIP3, a molecular mediator which promotes mitophagy, the antioxidant augmenter of liver regeneration (ALR), and the anti-apoptotic molecule BCL-2 in cancer cells of type I endometrial carcinoma with previously described alterations in respiratory complex I (oncocytic-like phenotype), as compared to matched non-malignant tissue and hyperplastic tissue, linking mitochondrial dysfunction to the expression of pro-fission, anti-oxidant, and anti-apoptotic proteins [24].

Tanwar et al. conducted experiments of downregulation of DRP1 in a human ovarian carcinoma cell line, showing a potential causal role of DRP1 in mitotic transition and cell proliferation in EOC cells [74]. These authors have also compared the expression of aldehyde dehydrogenase 1A1 (Aldh1A1), a marker for ovarian cancer stem cells, between primary and relapse tumor samples and have found an inverse relationship between Aldh1A1 and DRP1 expression [74]. This finding suggests that the modulation of DRP1 may potentially be involved in the stem cell properties of the relapsed EOC disease [74]. Based on their results, DRP1 seems to associate with cell cycle acceleration in some relapsed resistant patients (DRP1-High) as compared to others (DRP1-Low) where this does not seem to happen. The authors thereby hypothesize that DRP1 may have a pro-apoptotic role in DRP1-Low and an anti-apoptotic role in DRP1-High patients [74]. Additionally, they have suggested that a DRP1-based-gene expression-signature from primary tumors could stratify patients for survival after exposure to chemotherapy, since the pattern of genes expression seems to differ in both DRP1-High and DRP-Low groups [74].

The RAS-activated molecule recombinant protein of human ralA binding protein 1 (RALBP1) regulates the effect of Cyclin B1 on DRP1 [54,55]. Although RAS-ERK signaling-driven regulation of DRP1 contributes to cell transformation, as previously mentioned, no relationship with cell cycle alteration was found [19,59]. Various studies have implicated extracellular signal-regulated kinase 1 and 2 (ERK1/2) in regulating DRP1 function (Figure 1). Yu et al. have shown that ERK1 could phosphorylate DRP1 in vitro [103]. Gan et al. studied the oxidative stress responses in cytoplasmic hybrid (cybrid) derivatives of neuronal cells, incorporating platelet mitochondria from AD [104]. They showed that ERK1/2 activation driven by oxidative stress increased DRP1 expression and its recruitment to mitochondria, generating increased fission in AD cybrids [104]. However, no functional link between ERK and DRP1 was established [104]. As mentioned previously, Serasinghe et al. have demonstrated that DRP1^S616^ is phosphorylated by ERK1/2 in cancer cells, promoting mitochondrial fission to support RAS-dependent transformation and tumor growth [19]. When this phosphorylation was reverted in vitro, cells have undergone apoptosis [19]. Recently, Kashatus et al. showed that the expression of mutant *RAS* in HEK cells promoted DRP1-dependent mitochondrial fragmentation [61]. Additionally, knockdown of *DNM1* inhibited the growth of transformed cell tumor xenografts [61]. ERK2 and activated RAS, RAF or MEK mutants were shown to phosphorylate human DRP1^S616^ in vitro, an effect that was abolished by MEK inhibitors [61]. This was accompanied by a reversal of the mitochondrial fission [61].

ERK1/2-dependent DRP1 phosphorylation and mitochondrial fission have been described to induce pluripotent stem cells (iPSCs) during the reprogramming of somatic cells [105]. Prieto et al. have shown that cellular reprogramming into iPSC induced mitochondrial fission early in this process, which was dependent on DRP1 and accompanied by an increase in DRP1 phosphorylation at the murine equivalent of human DRP1^S616^, with kinetics matching DRP1 recruitment to mitochondria [106]. It was also shown that mitochondrial fission was inhibited by a MEK inhibitor, a pattern which was reverted by a DRP1 phosphomimetic mutant. This raised the hypothesis that ERK signaling may be required for mitochondrial fission early in the reprogramming process [106].

Morita M. et al. have shown that the nutrient-sensing mechanistic/mammalian target of rapamycin complex 1 (mTORC1), which is known to be activated in many different malignant tumors, stimulates the translation of mitochondrial fission process 1 (MTFP1) protein [107]. MTFP1 is, in its turn, associated with phosphorylation and mitochondrial recruitment of DRP1 and a mitochondrial fission pattern [107]. Interestingly, they have found that the suppression of mTORC1 activity led to increased mitochondrial fusion due to the reduced translation of MTFP1, which is mediated by translation initiation factor 4E (eIF4E)- binding proteins (4E-BPs) [107]. The authors further concluded that uncoupling MTFP1 levels from the TORC1/4E-BP pathway after mTOR inhibition blocks the hyperfusion status and leads to apoptosis, thereby offering a new therapeutic opportunity for these type of anti-cancer drugs, converting them from cytostatic to cytotoxic [107].

The mitochondrial uncoupling protein 2 (UCP2) also seems to control mitochondrial fission through DRP1 expression regulation [108,109]. Toda et al. reported mitochondrial changes, such as increase in mitochondrial density and reduction in mitochondrial size, in ventromedial nucleus of the hypothalamus (VMH) neurons mediated by UCP2, suggesting that UCP2 is involved in the regulation of the mitochondrial fission process [110]. In this way, Toda et al. assessed the effect of UCP2 in DRP1 in response to a glucose load and verified a significant increased ratio of phosphorylated DRP1/DRP1 in UCP2 knockout mice with selective re-expression of UCP2 [110]. Interestingly, a few years ago, UCP2 was found to be overexpressed in Hürthle cell tumors [111]. These findings may partially explain the pattern of DRP1 overexpression observed by Silva et al. in Hürthle cell tumors of the thyroid, known to be characterized by at least 75% of oxyphilic cells [23].

## 7. Role of Mitochondrial Dynamics in Invasion and Metastization

In a series of human breast cancer samples, Zhao et al. observed a significantly increased expression of DRP1 protein in in situ ductal carcinoma in comparison with normal tissue, and in invasive breast cancer and lymph node metastases in comparison with in situ ductal carcinoma [21]. The authors also reported an increased expression of DRP1 and phosphorylated DRP1^S616^ in metastatic breast cancer cell lines, as compared to non-metastatic breast cancer cell lines [21]. DRP1 genetic inhibition led to reduced migration and invasion capacities, which was also verified for cell migration when pharmacological inhibition with Mdivi-1 was tested [21]. Cell cycle or cell viability did not seem to be affected by DRP1 changes [21]. Interestingly, DRP1 silencing led to reduced cell spreading and lamellipodia formation, typically seen in the edge of migrating cells, which was accompanied by a change in mitochondria distribution within the cell, from perinuclear to a more scattered state, independent of the membrane potential [21]. The aforementioned findings suggested that the upregulation of DRP1 may be an early event in invasive breast cancer development, and formation of lamellipodia is dependent of mitochondria fission [21].

It was demonstrated in a glioblastoma in vitro model that hypoxia induces upregulation of DRP1, mitochondrial fission and cell migration [112,113,114,115]. Following these observations, Han et al. looked at the effect of hypoxia in breast cancer cell migration driven by mitochondrial dynamics [20]. Besides the similar pattern of DRP1 expression in metastatic breast cancer cell lines documented before, Han et al. showed that hypoxia led to mitochondrial fission and to a significantly increase in migration of the metastatic cell line in comparison with the non-metastatic cell line. The genetic inhibition of DRP1, as well as the used of Mdivi-1, led to a significant reduction in mitochondrial fission as well as in hypoxia-induced migration [20]. At variance with the non-metastatic cell line, treatment with cisplatin (CDDP) induced apoptosis, mitochondrial fission, increase in intracellular levels of ROS and a decrease in metalloproteinase (MMP) in the metastatic cell line, which was reverted by the inhibition of DRP1 [96]. These results indicate that mitochondrial fission driven by DRP1 induces the metastatic cell line to become more sensitive to cisplatin in hypoxic conditions, potentially but not only through the impact on intracellular ROS and MMP, an effect that was not observed in the non-metastatic cell line [96].

Finally, a study that has shed some light onto the mechanisms that link cell motility and migration with mitochondria and OXPHOS dysfunction, has been published by our group [116]. We have shown that cybrid cells harboring a specific mtDNA mutation are less prone to apoptosis, have a higher motility and migration ability, and produce larger tumors and more lung metastases in a mouse model in comparison with wild-type cells [116].

## 8. Future Perspectives and Clinical Implications

**Figure 2:**
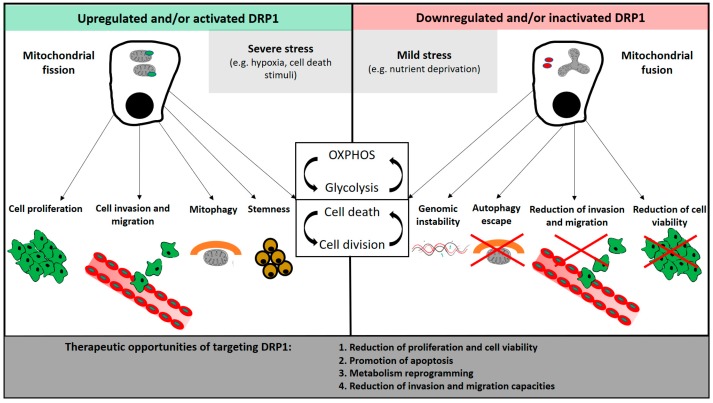
Effects of DRP1 activation and/or upregulation, and associated mitochondrial fission patterns, on tumorigenesis. Inactivation and/or downregulation of DRP1 may have a counteracting effect on tumorigenesis, which could be used as a therapeutic approach in cancer. The effects of both DRP1 activation and inactivation on metabolism reprogramming, and on cell cycle and cell death, should be seen as a continuously dynamic adaptive mechanism to internal and external challenges.

The role of DRP1 in key hallmarks of cancer, as cell proliferation and survival, apoptosis failure, metabolic reprogramming, invasion and metastization, and even insensitivity to anti-growth or anti-proliferative signals, depends most likely from the interplay between microenvironment stimuli, cells’ genetic background, cytotoxic or targeted treatment strategies, and the tumor cell’s continuous adaptation to all of these factors. In other words, we may look at DRP1 as a key molecular link between several biological cell processes, which acts as a key player in the plasticity of tumoral cells under various internal and external contexts (Figure 2). This concept has implications both on the interpretation of its biological significance at any given moment of the tumorigenesis process, as well as on the potential effects of its inhibition which can also be paradoxical. As an example, Szabadaki et al. have shown that DRP1 overexpression can prevent apoptosis, but it had a negative effect on cancer survival following MAPK inhibitors [19]. There is evidence suggesting that some tumor cells may become dependent on ERK1/2-driven DRP1 phosphorylation, thus indicating that DRP1 inhibition may be a potential therapeutic strategy for such tumors [104]. Others, however, have demonstrated that DRP1 inhibition can prevent cell death and promote proliferation [29,65,66].

Some of the research presented in this revision suggest a new concept, in which mitochondrial-targeted cancer therapy could be additive to or synergized with therapies that address cancer cell proliferation, such as promoting mitochondrial glucose oxidation [19].

It remains important that the link between DRP1 and cell cycle is better understood. Mitra et al. have found that the G_1_–S transition and Cyclin E levels can be regulated by the mitochondrial state, thereby opening new areas of exploration relating mitochondria with cancer [29]. Zou et al. have stressed the emerging evidence of PGC1α contributing to tumor growth, and therefore have proposed the critical importance to target both mitochondrial biogenesis and mitophagy for effective cancer treatment, a concept to be tested in future research as a means to test effectiveness for breast cancer treatment [68]. Additionally, the definition of a relationship between HIF-1α and DRP1 may be of relevance to assess its clinical applications in the future [71].

Finally, we believe it is worthwhile to stress the research recently published by Tanwar et al. [74]. Their DRP1-based analysis highlights that DRP1-driven cell cycle regulation is present in several cancer types, which may allow response to therapies targeting proliferating cells [74]. In particular, their results point out to an important role of mitochondria in ovarian cancer chemo-resistance and relapse [74].

To address the issue on how DRP1 can be targeted, it is important to highlight that, although Mdivi-1 has been widely used as a putative DRP1 inhibitor in vitro and in vivo, including in much of the published data referenced in this review, a recent report has proposed an alternative mechanism of action for this compound, as a reversible mitochondrial complex I inhibitor, not impairing Drp1 GTPase activity. Targeting DRP1 in the context of cancer still seems a promising approach, but not without the challenges of designing and developing compounds that specifically inhibit GTPase activity, and of the complex interplay between mitochondria dynamics and cell requirements in every stage of tumorigenesis.

## Figures and Tables

**Table 1:** Summary of Dynamin-related protein 1 (DRP1) interplay with key cellular processes.

Cell Process	Effects
**Cell Death**	DRP1 associates with bcl-2-associated X protein (BAX) at mitochondrial fission sites, promoting permeabilization of the outer mitochondrial membrane (OMM) and cytochrome *c* release [64]
	DRP1 drives balance between fission-fusion impacting mitochondrial Ca^2+^ responses in apoptotic signaling [65]
	DRP1 inhibition inhibits BAX-BAK dependent cytochrome *c* release [66]
	DRP1 knockdown reduces caspase-3 activation and apoptosis [67]
	DRP1 inhibition is associated with increase in apoptosis [22]
**Metabolic Reprogramming**	DRP1 upregulation associates with less metabolically active mitochondria and increased mitochondrial biogenesis [68]
	DRP1 inhibition associates with increased mitochondria oxidative capacity [68]
	**Response to hypoxic conditions:**
	DRP1 expression increased [69]
	DRP1 expression decreased after inhibition of HIF-1α [69]
	DRP1 inhibition affects HIF1-α expression [69]
	**Response to starvation:**
	Decrease in mitochondrial fraction and activation of DRP1^S616^ through PKA activation [53,70]
	Elongation of mitochondria [70]
	Shift from glycolysis to oxidative phosphorylation (OXPHOS) [70]
	Activation of LDH-A and PDK1 HIF-1α target genes [70]
	OXPHOS/glycolysis interchange through HIF-1α /c-MYC pathway [71]
**Cell Cycle**	DRP1 functionally or molecularly linked to Cyclin B, E and D [19,29,54,55,72,73]
	DRP1 correlates with cell-cycle genes in various cancer types [74]
	Mitochondrial morphology is associated with cell cycle control at the G1–S boundary [29,54]
	DRP1 inhibition is associated with decrease of cell viability and mitotic program [29,54]
	DRP1 knockdown reduces proliferation and percentage of cells in sub-G_0_/G_1_ cell cycle phase [67]
	DRP1 downregulation associates with activation of DNA damage signaling pathways and ATM kinase-dependent G_2_/M cell cycle checkpoint, genomic instability and aneuploidy [28]
	DRP1 inhibition significantly decreases tumor size [22]

BAX: Bcl-2-associated X protein; BAK: Bcl-2-associated death promoter protein; HIF1-α: hypoxia-inducible factor 1; PKA: protein kinase A; LDH-A: lactate dehydrogenase A; PDK1: pyruvate dehydrogenase kinase 1; c-MYC: myelocytomatosis oncogene protein; ATM: ataxia telangiectasia mutated protein.

**Table 2:** Summary of DRP1 expression patterns and tumorigenic effects in different tumor models.

Tumor Model	DRP1 Expression Pattern and Tumorigenic Effects
**Melanoma**	Expression of phosphorylated DRP1^S616^ associated with *BRAF^V600E^* pre-neoplastic lesions and malignant tumors [95]
**Lung Cancer**	Overexpression of DRP1 ex vivo [22] Increased expression of phosphorylated DRP1^S616^ and decreased levels of phosphorylated DRP1^S637^ in vitro [22]
**Breast Cancer**	DRP1 expression associated with invasive tumors and lymph node metastases ex vivo [21,96] Expression of phosphorylated DRP1^S616^ in vitro [21,96] Invasion and migration capacities in vitro, including hypoxia-induced [21,96]
**Thyroid Cancer**	Overexpression of DRP1 in oncocytic tumors and oncocytic carcinomas ex vivo [23] Invasion and migration in vitro [23]
**Type I Endometrial Cancer**	DRP1 expression ex vivo associated with mitochondrial dysfunction, anti-apoptotic and anti-oxidant profile [24]
**Epithelial Ovarian Cancer**	DRP1 expression ex vivo associated with anti-apoptotic profile [74] DRP1 driven mitosis linked to chemo-sensitivity of primary tumors [74]
**Neuroblastoma**	DRP1 expression and mitochondrial translocation in vitro associated with Survivin anti-apoptotic effects and glycolytic phenotype [97]
**Glioblastoma**	Upregulation of DRP1 and hypoxia-induced cell migration in vitro [98]
